# Clinical outcome of PSMA-guided radiotherapy for patients with oligorecurrent prostate cancer

**DOI:** 10.1007/s00259-020-04777-z

**Published:** 2020-05-13

**Authors:** Stefan A. Koerber, Katharina Sprute, Clemens Kratochwil, Erik Winter, Matthias F. Haefner, Sonja Katayama, Ingmar Schlampp, Klaus Herfarth, Klaus Kopka, Ali Afshar-Oromieh, Stefanie Zschaebitz, Tim Holland-Letz, Peter L. Choyke, Dirk Jaeger, Markus Hohenfellner, Uwe Haberkorn, Juergen Debus, Frederik L. Giesel

**Affiliations:** 1 Department of Radiation Oncology, Heidelberg University Hospital, Im Neuenheimer Feld 400, 69120 Heidelberg, Germany; 2 National Center for Tumor diseases (NCT), Heidelberg, Germany; 3 Heidelberg Institute of Radiation Oncology (HIRO), Heidelberg, Germany; 4 Department of Nuclear Medicine, Heidelberg University Hospital, Heidelberg, Germany; 5 Clinical Cooperation Unit Nuclear Medicine, German Cancer Research Center (DKFZ), Heidelberg, Germany; 6 Heidelberg Ion-Beam Therapy Center, Department of Radiation Oncology, Heidelberg University Hospital, Heidelberg, Germany; 7 Division of Radiopharmaceutical Chemistry, German Cancer Research Center (DKFZ), Heidelberg, Germany; 8 Institute of Radiopharmaceutical Cancer Research, Helmholtz-Zentrum Dresden Rossendorf (HZDR), Dresden, Germany; 9 German Cancer Consortium (DKTK), partner site, Heidelberg, Germany; 10 Department of Nuclear Medicine, University Hospital Bern, Bern, Switzerland; 11 Department of Medical Oncology, Heidelberg University Hospital and National Center for Tumor Diseases (NCT), Heidelberg, Germany; 12 Department of Biostatistics, German Cancer Research Center (DKFZ), Heidelberg, Germany; 13 Molecular Imaging Program, Center for Cancer Research, National Cancer Institute, National Institutes of Health, Bethesda, MD USA; 14 Department of Urology, Heidelberg University Hospital, Heidelberg, Germany; 15 Clinical Cooperation Unit Radiation Oncology, German Cancer Research Center (DKFZ), Heidelberg, Germany

**Keywords:** Prostate cancer, PSMA, PET, Metastases, SUV, OS

## Abstract

**Purpose:**

First-line treatment of patients with recurrent, metastatic prostate cancer involves hormone therapy with or without additional systemic therapies. Prostate-specific membrane antigen (PSMA) positron emission tomography (PET)/computed tomography (CT) allows the detection of oligometastatic disease that may be amenable to image-guided radiotherapy. The current study classifies the type and localization of metastases and the clinical outcome of PSMA-PET/CT-guided radiotherapy to selected metastases.

**Materials and methods:**

Between 2011 and 2019, 86 patients with recurrent, oligometastatic prostate carcinoma were identified by PSMA-PET/CT and were treated with image-guided radiotherapy of their metastases. Sites of relapse were characterized, and the primary endpoint overall survival (OS), biochemical progression-free survival (bPFS), and androgen deprivation therapy (ADT)-free survival were tabulated.

**Results:**

In total, 37% of the metastases were bone metastases, 48% were pelvic nodal metastases, and 15% were nodal metastases outside of the pelvis. After PSMA-guided radiotherapy, a biochemical response was detected in 83% of the cohort. A statistically significant decrease in the standard uptake value (SUV) was seen in irradiated metastases. After a median follow-up of 26 months, the 3-year OS and bPFS were 84% and 55%, respectively. The median time of ADT-free survival was 13.5 months. A better clinical outcome was observed for patients receiving concomitant ADT or more than 24 fractions of radiation.

**Conclusion:**

PSMA-guided radiotherapy is a promising therapeutic approach with excellent infield control for men with oligorecurrent prostate carcinoma. However, prospective, randomized trials are necessary to determine if this approach confers a survival advantage.

## Introduction

In 1995, Hellman and Weichselbaum coined the phrase “oligometastases” to describe a state of cancer with a limited number of metastases in only one or a few sites [1]. From that time, numerous studies advocated for a local, aggressive treatment approach of patients with oligometastatic disease. An open-label phase 2 study randomized a cohort of 99 patients with a controlled primary tumor and 1–5 metastatic lesions to either palliative standard treatment or standard of care plus local treatment of metastases using stereotactic body radiation therapy (SBRT). After a median follow-up of 25 months, SBRT was associated with an improved overall survival compared with the control group [2]. Metastasis-directed therapy is even integrated in clinical guidelines for several tumor types. However, there is a lack of data regarding treatment of oligometastatic prostate carcinoma. The prostate-specific membrane antigen (PSMA) positron emission tomography (PET)/computed tomography (CT) enables detection of small metastases hitherto undetectable by conventional imaging [3–7]. Recently, Ost et al. reported a significantly longer androgen deprivation therapy (ADT)-free survival for a cohort of 62 patients with oligorecurrent prostate cancer undergoing metastasis-directed therapy (MDR) compared with surveillance alone. Although the study was planned as a prospective, multicenter phase 2 trial, the number of patients was limited and no data were available on overall survival (OS) [8]. To this end, we aimed to evaluate the clinical outcome after local SBRT for patients with oligorecurrent prostate carcinoma detected by PSMA-PET/CT imaging.

## Materials and methods

### Patient cohort

At the Department of Nuclear Medicine, Heidelberg University Hospital, more than 2500 men with prostate cancer underwent PSMA-PET/CT imaging between July 2011 and December 2018. Inclusion criteria for this study included sufficient clinical data, recurrent prostate cancer after primary therapy, and PSMA-PET/CT imaging with oligometastatic disease defined as a maximum of 5 metastases. From patients, 86 met the inclusion criteria.

### PSMA-PET/CT imaging

Imaging was performed with a ligand of PSMA using three different scanners: 63 patients (73.2%) were scanned with a Biograph mCT Flow scanner while a Biograph 6 PET/CT scanner was used for 21 patients (24.7%). Two patients (2.3%) underwent scanning with a Biograph 20mT scanner. The average activity used for the imaging was 225.93 (± 52.7) MBq with a range from 77 to 325 MBq. For 74 patients (86.0%), ^68^Ga-PSMA-11 was used as a tracer, while for 12 patients (14.0%), PSMA imaging was performed using ^18^F-PSMA-1007. Mean acquisition time was 68.5 min (± 30.2 min). In the course of the imaging, the standard uptake value (SUV) was measured for each metastatic lesion. All scans were performed and interpreted as previously published [9, 10].

### Follow-up

Clinical follow-up was performed 6–8 weeks after radiotherapy and then regularly according to national guidelines (every 3 months in the first 2 years followed by every 6 months for the next 2 years and then once a year) including a measurement of the prostate-specific antigen (PSA) level and urological examination. Furthermore, toxicity and side effects were evaluated during and after radiotherapy using the Common Terminology Criteria for Adverse Events (CTCAE) version 5.0.

### Statistical analysis

Data were analyzed using the statistical program IBM SPSS Statistics version 25. The primary endpoints were defined as overall survival (OS), biochemical progression-free survival (bPFS), and androgen deprivation therapy (ADT)-free survival. Biochemical failure was defined as (1) a raise of the serum PSA over 2 ng/ml above PSA nadir after definitive radiotherapy and (2) two consecutive rises of PSA from nadir after primary radical prostatectomy. As secondary outcomes, the localization of the metastases, acute and chronic toxicity, and quality of life at the time of follow-up was evaluated using the European Organization for Research and Treatment of Cancer quality of life questionnaire-core 30 (EORTC QLQ-C30) version 3.0.

The Kaplan-Meier method was used to estimate OS, bPFS, and ADT-free survival. Furthermore, the two-sided Wilcoxon test was used to compare two different groups or measurements. Results were defined as statistically significant if the *p* level was less than 0.05.

## Results

### Patient characteristics and treatment

All 86 patients underwent PSMA-PET/CT imaging for PSA relapse after primary treatment. Seventy-four patients (86.0%) presented with recurrent disease after surgery while 11 patients (12.8%) presented after definitive radiotherapy. Overall, the patient’s initial disease was classified as high-risk according to the d’Amico risk classification in 94.0% [11] (Table 1).

**Table 1:** Patient characteristics

Characteristics		
Age at initial diagnosis (*n* = 86)		
	Median (range) (years)	65 (49–80)
Initial PSA value (*n* = 74)		
	Median (range) (ng/ml)	10.7 (2.8–520)
Gleason score (*n* = 79)		
	6	2 (2%)
	7	40 (50.6%)
	8	8 (10.1%)
	9	27 (34.2%)
	10	2 (2.5%)
Risk group according to d’Amico (*n* = 83)		
	Low	1 (1.2%)
	Intermediate	4 (4.8%)
	High	78 (94.0%)
Initial treatment (*n* = 86)		
	Radiotherapy	11 (12.8%)
	Surgery	74 (86.0%)
	Other	1 (1.2%)
First/second relapse (*n* = 81)		
	First relapse	52 (64.2%)
	Second relapse	29 (35.8%)

PSMA-PET/CT detected 168 PSMA-positive lesions in 86 patients (mean 1.96 per patient). Only one metastasis was seen in 54% of patients (Table 2). A total of 25.9% of the patients presented 2 metastases; 3 or more than 3 metastases were visible in 20.1% of the cohort. Pelvic nodal metastases were the most frequent site of oligometastatic disease (48.2%). The mean time between PSMA-PET/CT and the start of MDR was 55.4 days. For radiotherapy planning, all pathological findings on PSMA-PET/CT were defined as the gross target volume. Clinical target volume was obtained by adding a margin of 2–3 mm.

**Table 2:** Results of PSMA-PET/CT

Characteristics		Number of patients
PSA value at the time of PET/CT (*n* = 77)		
	Mean ± SD (ng/ml)	3.0 ± 4.5
	Median (range) (ng/ml)	1.2 (0.03–25)
Number of metastases (*n* = 85)		
	Mean ± SD	1.9 ± 1.6
	Median (range)	1.0 (1–5)
Localization of metastases		
	Total number of metastases	168
	Bone	62 (36.9%)
	Pelvic nodes	81 (48.2%)
	Distant nodes	25(14.9%)

An additional margin of 2–3 mm was given to define the planning target volume. For elective node irradiation (ENI), a margin of 5 mm was chosen for planning target volume. Forty-four patients (51.2%) underwent ENI and 52 of 84 patients (61.9%) received ADT at the time of radiotherapy. Irradiation was performed using helical intensity-modulated radiotherapy (IMRT) in 77 patients (90.6%). Other irradiation techniques included 3D conformal radiation therapy or CyberKnife. The most commonly fractionation schedules were 27.0 gray (Gy) in 3 fractions and 37.5 Gy in 5 fractions for local irradiation as well as 51.0 Gy in 34 fractions for ENI including a simultaneous integrated boost to the PSMA-positive lesion (61.2 Gy or 57.2 Gy). In total, 51 patients (59.3%) received more than 24 fractions, 21 patients (24.4%) were irradiated with 10 to 24 fractions, and 14 patients (16.3%) received fewer than 10 fractions.

### Clinical outcome

**Fig. 1:**
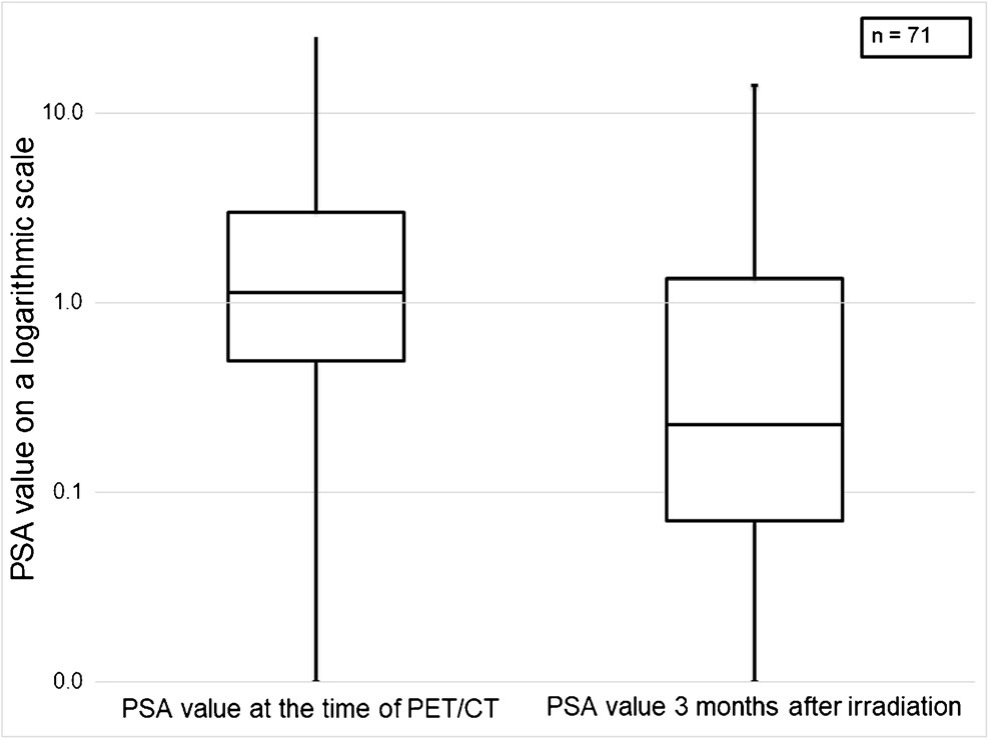
PSA profile before and after radiotherapy. Boxplot of PSA-values

**Fig. 2:**
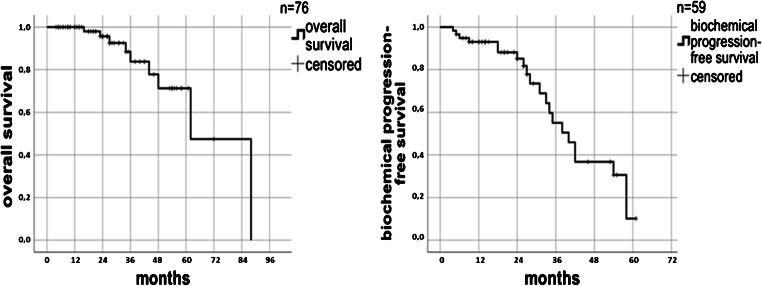
Kaplan-Meier estimates of overall survival (left) and biochemical progression-free survival (right)

A PSA decrease occurred in 59 out of 71 patients (83.1%) at 3 months after PSMA-guided radiation (Fig. 1). For 7.0%, PSA did not change after radiotherapy while for 9.9%, the PSA value continued to rise. In the case of a PSA decrease, the value declined over 50% in 73.3% of the cohort; the median reduction of the PSA value was 75.54%. The Wilcoxon test reveals the difference as significant (*p* < 0.001). Patients, in whom the PSA value increased after the irradiation (*n* = 7), showed more PSMA-positive lesion per patient than the overall cohort: mean 2.57 vs. 1.96, in terms of the initial PSA value or the Gleason score, there was no discrepancy. After a median follow-up of 26 months (range 4–72 months), 8 of 86 men were lost to follow-up. In the remaining 78 patients contacted for a follow-up visit, 60 patients attended the appointment. In total, 69 of 78 patients (88.4%) were still alive. The 2-year and 3-year OS rates were 95.7% and 83.7%, respectively. A biochemical failure after PSMA-guided radiotherapy occurred in 21 patients (35.6%) at the time of follow-up leading to a 2-year and 3-year bPFS of 85.1% and 55.1% (Fig. 2). The median time of ADT-free survival was 13.5 months. In subgroup analyses, patients receiving concomitant ADT during radiotherapy had a statistically significant lower risk for death or relapse compared with patients not on systemic therapy (*p* = 0.001). Similar results were observed with regard to the number of treatment fractions: Patients receiving more than 24 fractions had improved survival or clinical response compared with those receiving 9 or fewer fractions (*p* = 0.042).

### PSMA-response

**Fig. 3:**
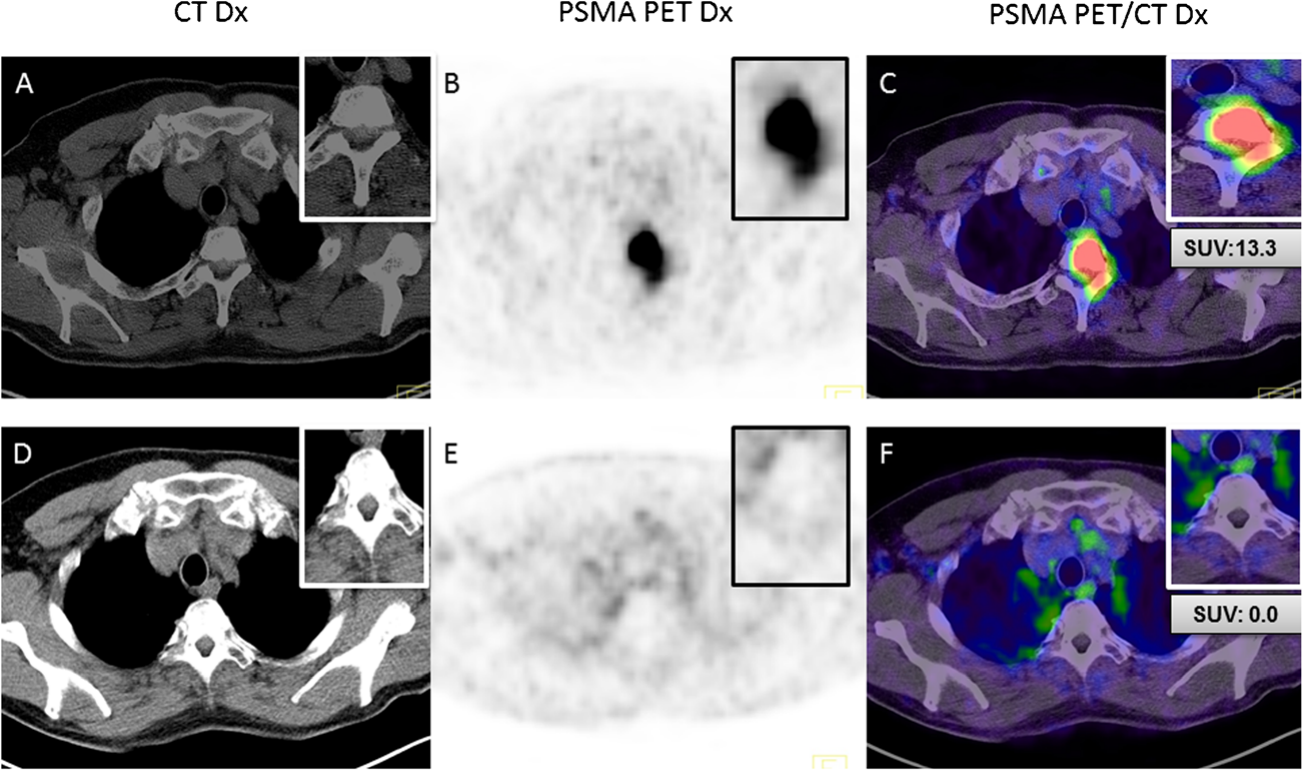
64-year old prostate cancer patient with a bone metastasis in T2, before PSMA-guided radiotherapy (**a**–**c**; SUVmax 13.3) and after irradiation (**d**–**f**; SUVmax 0)

**Fig. 4:**
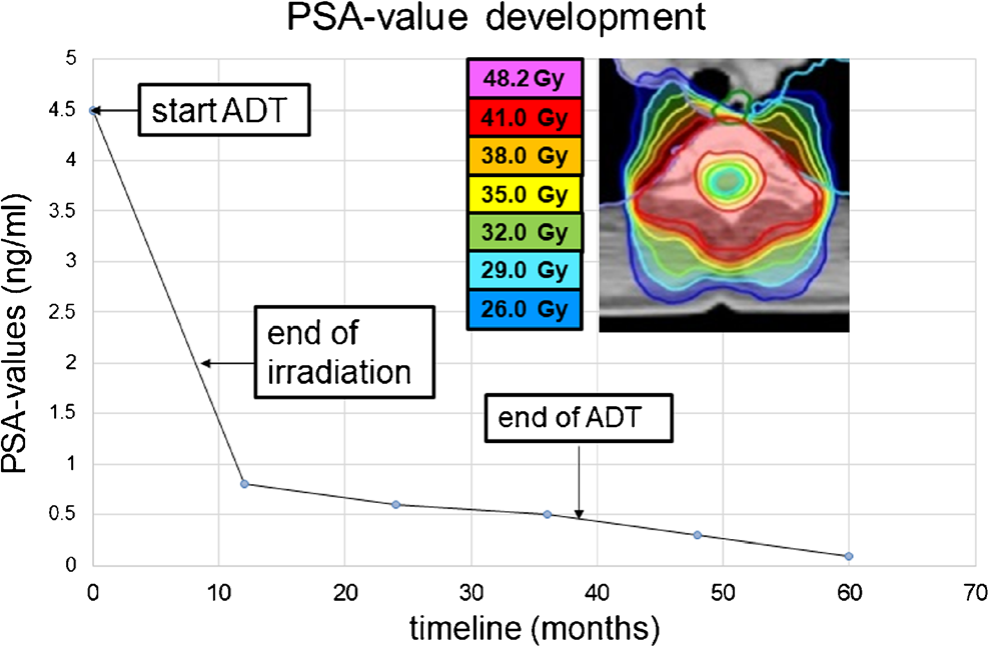
Corresponding PSA-values after local irradiation with PSA response followed by biochemical progression in 12/201. ADT, androgen deprivation therapy

After irradiation, a PSMA-PET/CT was available for 28 patients (32.6%) with a median interval of 13 months (range 4–43 months). For almost all patients, hybrid imaging was performed for restaging. The SUV of 38 lesions was compared before and after the irradiation. Tumor lesions had a median standardized uptake value SUVmax of 7.45 (range 1.7–69) before irradiation which decreased to a median SUVmax of 0 (range 0–27.3) after irradiation (Figs. 3 and 4). In total, PSMA-guided radiotherapy resulted in a lesion-based reduction of the mean SUVmax of 81.9% (*p* < 0.003); however, in the same interval, an average of 2.7 new PSMA-positive lesions per patient was found. Local control was 90.9%; two patients demonstrated relapse within the former target volume (prostate bed [one patient] and iliac node [one patient]).

### Toxicity

Before irradiation, 59.3% of all men reported mild (grade 1/2) genitourinary (GU) symptoms after primary therapy, while grade 3 GU toxicity was seen in 2.3%. During radiotherapy, an increase of GI- and GU toxicity was observed (grade 1/2; 33.0% [GI], 62.0% [GU]). The most common side effects were diarrhea, urinary frequency, and deterioration of urinary continence. Furthermore, grade 1 and 2 fatigue occurred more frequently (23.8% and 1.2%, respectively). However, after 3 months, the symptoms had mostly resolved with the most significant toxicity observed for GU side effects (23.3%) (Table 3). Differences of quality of life according to EORTC QLQ-C30 were observed especially in functional (e.g., role function) and symptomatic fatigue, dyspnea, and sleeping problems. Nevertheless, the global health status of the metastasized patients showed a mean of 66.9 scoring points which is comparable with the score of the healthy population of men at this age (71.6) [12].

**Table 3:** Overview of toxicity

Characteristics		Before RT (%)	During RT (%)	3 months after RT (%)	Last follow-up (%)
GI toxicity					
	Grade 0	91.9	67.1	84.2	53.3
	Grade 1	8.1	30.6	14.5	35.0
	Grade 2	-	2.4	1.3	8.3
	Grade 3	-	-	-	3.3
GU toxicity					
	Grade 0	38.4	29.8	30.3	3.3
	Grade 1	41.9	31.0	40.8	35.0
	Grade 2	17.4	31.0	25.0	38.3
	Grade 3	2.3	8.3	3.9	23.3
Edema					
	Grade 0	97.7	92.8	93.4	60.0
	Grade 1	2.3	7.2	6.6	26.7
	Grade 2	-	-	-	10.0
	Grade 3	-	-	-	3.3
Fatigue					
	Grade 0	98.8	75.0	80.3	31.7
	Grade 1	1.2	23.8	17.1	38.3
	Grade 2	-	1.2	2.6	26.7
	Grade 3	-	-	-	3.3

*GI*, gastrointestinal; *GU*, genitourinary; *RT*, radiotherapy

## Discussion

The clinical outcome of patients with metastatic prostate cancer improved with intensified systemic therapy. Several large, prospective trials reported enhanced overall survival with docetaxel or abiraterone acetate plus prednisone in men with newly diagnosed, metastatic disease [13–16]. However, all these trials were done before PSMA-PET/CT was widely available. Due to its high sensitivity, PSMA imaging is often performed in patients with PSA relapse after primary treatment leading to an increased number of patients diagnosed with oligorecurrent disease. Such lesions are invariably invisible on conventional CT or bone scan. While it would be logical that patients with oligometastatic disease might have better outcomes than patients with widespread metastases and might be more responsive to therapy, this hypothesis has not yet been proven.

For men with oligorecurrent prostate cancer, MDR may offer a new treatment approach delaying disease progression and the toxicities of systemic therapy. While several studies report on clinical outcome of local irradiation based on “conventional” imaging like MRI or choline PET (Table 4), only few studies of PSMA-guided MDR are available. In the current trial, a PSA response occurred in 83.1% after local irradiation of oligorecurrent prostate cancer patients who underwent PSMA-PET/CT. This is comparable with the finding of a study of 83 patients with biochemical recurrence after surgery wherein PET-guided, fractionated radiotherapy of nodal relapses decreased PSA in 82.9% of patients [22]. Interestingly, the excellent local control rates after irradiation were strongly correlated with a decrease of SUVmax in those patients undergoing PSMA imaging during follow-up. For all patients in our cohort, a statistically significant decrease of tracer uptake after irradiation was observed confirming that PSMA-PET/CT imaging may also aid in the assessment of treatment response. Even though we detected a PSA rise for a certain number of patients during follow-up, bPFS was quite high in our cohort. In contrast, 43.5% biochemical progression was reported in a study of 108 men with recurrent prostate carcinoma and PSMA-guided, normo-, or moderate hypofractionated radiotherapy after a median follow-up of 18 months [23]. Furthermore, a prospective trial of 33 oligorecurrent patients undergoing stereotactic ablative body radiotherapy demonstrated a 1- and 2-year disease PFS of 58% and 39%, respectively [24]. These differences may relate to differences in radiation planning and the location of metastases.

**Table 4:** Overview of trials evaluating the role of metastasis-directed therapy without the use of PSMA-PET/CT

Characteristics	Number of patients	Status of disease	Imaging/treatment	Median follow-up time	Outcome
Jereczek-Fossa et al. [17]					
	34 (38 lesions)	Relapse (15 patients with local relapse only)	Bone scan, CT, choline PET/CT/ 30 Gy (5 fx) or 33 Gy (3 fx) or 36 Gy (3 fx)	16.9 months	30 months PFS 42.6%; LC 35 out of 38 lesions
Decaestecker et al. [18]					
	50 (70 lesions)	Relapse (up to 3 synchronous metastases)	FDG or choline PET/CT/50 Gy (10 fx) + 1 month ADT or 30 Gy (3 fx); re-irradiation allowed	2 years	Median PFS 19 months; median ADT-free survival 25 months
Muldermans et al. [19]					
	66 (81 lesions)	75.8% castrate-resistant disease	Conventional imaging/median 16 Gy (range 16–24 Gy; 1 fx) or 30 Gy (3 fx)	16 months	2-year LC 82%; 2-year bPFS 54%; 2-year OS 83%
Bouman-Wammes et al. [20]					
	43 (54 lesions)	Hormone-sensitive disease (< 5 metastases)	Choline PET/CT/30 Gy (3 fx) or 3 5Gy (5 fx) or 45 Gy (3 fx), re-irradiation allowed	2.6 years	PSA response 67.4%; median ADT-free survival 15.6 months
Triggiani et al. [21]					
	100 (139 lesions)	Oligorecurrent	Bone scan, CT, choline PET/CT/50 Gy (10 fx), re-irradiation allowed	20.4 months	2-year LC 92.8%; 2-year dPFS 43%; 2-year ADT-free survival 47.3%

*fx*, fractions; *ADT*, androgen deprivation therapy; *LC*, local control; *bPFS*, biochemical progression-free survival; *OS*, overall survival; *dPFS*, distant progression-free survival;

Even though several outcome data are available with regard to MDR based on conventional imaging (Table 4), a direct comparison with the current results should be done with caution. Due to the higher detection rate of PSMA-PET/CT, a clinical benefit of PSMA-guided irradiation might be expected; however, a final evaluation is very difficult considering the great heterogeneity of the different trials with regard to, e.g., treatment technique, fractionation, imaging technique, and the use of ADT. Moreover, it remains an open question whether there is a clinical benefit for ENI in comparison with local, small-volume SBRT for patients with nodal relapse. The current trial included a relatively large number of patients with nodal relapse (63.1%), and ENI was performed for 51.2%. In a multicenter analysis comparing outcome and toxicity of SBRT and ENI in a cohort of 506 patients with nodal oligorecurrent prostate cancer, fewer nodal recurrences were recorded after normofractionated, elective nodal irradiation. The authors concluded that ENI is preferred to SBRT in the treatment of nodal oligorecurrences [25]. Our study also demonstrated that patients obtaining more than 24 fractions—a subgroup which includes all men receiving ENI—had a lower risk for death or relapse. Similarly, we observed this clinical benefit for all patients receiving concurrent ADT. A recently published study by Kroeze et al. reported similar results with significantly improved biochemical recurrence-free survival rates after MDR due to the addition of ADT [26]. There is an urgent need for further studies assessing different strategies in radiation oncology for PSMA-guided radiotherapy.

Our study confirmed that PSMA-guided MDR is safe and well tolerated. While only a low rate of grade 3 GU toxicity was observed 3 months after radiotherapy, no grade 3 GI toxicity occurred nor did edema or fatigue. Lépinoy et al. reported on a similar grade 3 acute GU toxicity rate of 6.5% in a cohort of 62 patients with PET-positive nodal recurrence treated with either salvage-extended field radiotherapy or involved field radiotherapy. Similarly, no acute grade 3 or higher GI side effects were found. However, the rate of severe GU late toxicity in the French cohort was significantly below the rate in our trial (3.2 vs. 23.3%), even though one grade 4 event was recorded [27]. Overall, the rate of late toxicity was relatively high in the current study. The large proportion of men with symptoms and comorbidities before MDR as well as new therapies with potential side effects due to progressive disease in the follow-up period likely explains this difference. Furthermore, GU late toxicity in our cohort includes a large number of patients with sexual side effects (especially impotence and erectile dysfunction) which often occur in older men.

Although the current study is one of the largest evaluating the clinical role of PSMA-guided radiotherapy, it has several limitations. It is retrospective; the cohort there is a relatively short follow-up for patients with prostate carcinoma. Moreover, the number of men included in the current trial was relatively low which reduces the validity of subgroup analyses. The use of two different ligands may lead to some heterogeneity in the current trial. Even though the number of patients who underwent ^18^F-PSMA-1007-PET/CT was very low expecting no significant impact on the results of the current study, ^18^F-PSMA ligands may lead to improved detection rates within the urinary tract due to the low urinary clearance rate compared with ^68^Ga-PSMA [28]. However, our study demonstrated promising results of PSMA-guided MDR even though further research is required to identify optimal treatment regimens and specific patient’s characteristics leading to an oncological benefit.

### Conclusion

For men with oligorecurrent prostate cancer detected by PSMA-PET/CT imaging, individual, radiotherapeutic treatment approaches are safe and provide satisfactory clinical results. Even though the start of systemic therapy can be delayed by PSMA-guided radiotherapy, the best outcome was observed in patients with concomitant ADT. Moreover, ENI also demonstrated positive effects on men with nodal relapse. Furthermore, prospective trials are needed before PSMA-guided irradiation can be implemented into national guidelines.
